# Functional network disorganization and cognitive decline following fractionated whole-brain radiation in mice

**DOI:** 10.1007/s11357-023-00944-w

**Published:** 2023-09-25

**Authors:** Benjamin A. Seitzman, Francisco J. Reynoso, Timothy J. Mitchell, Annie R. Bice, Anmol Jarang, Xiaodan Wang, Cedric Mpoy, Lori Strong, Buck E. Rogers, Carla M. Yuede, Joshua B. Rubin, Stephanie M. Perkins, Adam Q. Bauer

**Affiliations:** 1 Department of Radiation Oncology, School of Medicine, Washington University in St. Louis, 4921 Parkview Place, Campus Box 8224, St. Louis, MO 63110 USA; 2 Mallinckrodt Institute of Radiology, School of Medicine, Washington University in St. Louis, 660 S. Euclid Ave, Campus Box 8225, St. Louis, MO 63110 USA; 3 Department of Biomedical Engineering, McKelvey School of Engineering, Washington University in St. Louis, St. Louis, MO USA; 4 Department of Psychiatry, School of Medicine, Washington University in St. Louis, St. Louis, MO USA; 5 Department of Pediatrics, School of Medicine, Washington University in St. Louis, St. Louis, MO USA

**Keywords:** Functional connectivity, Brain networks, Radiation therapy, Calcium imaging, Cognitive dysfunction

## Abstract

Cognitive dysfunction following radiotherapy (RT) is one of the most common complications associated with RT delivered to the brain, but the precise mechanisms behind this dysfunction are not well understood, and to date, there are no preventative measures or effective treatments. To improve patient outcomes, a better understanding of the effects of radiation on the brain’s functional systems is required. Functional magnetic resonance imaging (fMRI) has shown promise in this regard, however, compared to neural activity, hemodynamic measures of brain function are slow and indirect. Understanding how RT acutely and chronically affects functional brain organization requires more direct examination of temporally evolving neural dynamics as they relate to cerebral hemodynamics for bridging with human studies. In order to adequately study the underlying mechanisms of RT-induced cognitive dysfunction, the development of clinically mimetic RT protocols in animal models is needed. To address these challenges, we developed a fractionated whole-brain RT protocol (3Gy/day for 10 days) and applied longitudinal wide field optical imaging (WFOI) of neural and hemodynamic brain activity at 1, 2, and 3 months post RT. At each time point, mice were subject to repeated behavioral testing across a variety of sensorimotor and cognitive domains. Disruptions in cortical neuronal and hemodynamic activity observed 1 month post RT were significantly worsened by 3 months. While broad changes were observed in functional brain organization post RT, brain regions most impacted by RT occurred within those overlapping with the mouse default mode network and other association areas similar to prior reports in human subjects. Further, significant cognitive deficits were observed following tests of novel object investigation and responses to auditory and contextual cues after fear conditioning. Our results fill a much-needed gap in understanding the effects of whole-brain RT on systems level brain organization and how RT affects neuronal versus hemodynamic signaling in the cortex. Having established a clinically-relevant injury model, future studies can examine therapeutic interventions designed to reduce neuroinflammation-based injury following RT. Given the overlap of sequelae that occur following RT with and without chemotherapy, these tools can also be easily incorporated to examine chemotherapy-related cognitive impairment.

**Supplementary Information:**

The online version contains supplementary material available at 10.1007/s11357-023-00944-w.

## Introduction

Approximately 200,000 patients per year in the USA are treated with partial or whole-brain radiotherapy (RT) for primary or metastatic brain cancer. In adults, half of patients receiving intracranial irradiation survive long enough (6 or more months) to experience some form of cognitive dysfunction associated with RT [1, 2]. Beginning in as little as three months after RT, patients experience a decline in memory, followed by a progressive decline in other executive functions [3, 4]. Whole-brain RT is also commonly utilized in the treatment of pediatric brain tumors, such as medulloblastoma and CNS germinoma, and in the setting of brain tumors with spinal spread of disease. Since the majority of pediatric patients survive their diagnosis, there are numerous studies demonstrating long-term cognitive deficits in processing speed and executive function that persist into adulthood [5, 6]. While cognitive dysfunction is multi-factorial and related to (at least) tumor location, age at diagnosis, presence of hydrocephalus, and need for shunt, RT-specific factors correlating with poor cognitive outcome include total dose, fraction size, total volume of brain irradiated, temporal lobe dose, and hippocampal dose [7–9].

Despite the known toxicities of whole-brain RT, it remains an important treatment modality for both adult and pediatric brain tumor patients [10, 11]. Currently, the field is hampered because the mechanisms that underlie RT-related cognitive dysfunction are poorly understood. Studying the effects of RT on cognition in patients is further complicated since RT is rarely administered in isolation, and the presence of a tumor, seizures, and hydrocephalus all contribute to changes in cognitive function. In addition to radiation, patients are also commonly treated with surgery [12, 13], chemotherapy [14–16], and/or hormonal therapy [17, 18], all of which contribute to cognitive dysfunction. Therefore, pre-clinical models can provide insight into RT-specific alterations of brain and cognitive functions. While whole-brain RT has been demonstrated to significantly affect behavioral performance in mice, many animal studies fail to use a clinically-mimetic RT protocol, instead relying on high dose per fraction whole-brain protocols that substantially differ from human treatments [19, 20]. Data in the literature regarding behavioral deficits in mice receiving whole-brain radiation using traditional fractionation (1.8-3 Gy/fraction) are more limited.

A novel approach to studying RT-induced brain alterations in humans deploys functional magnetic resonance imaging (fMRI) to map network organization [21, 22]. Resting-state fMRI allows for measurement of network alterations in patients with a variety of neurologic and psychiatric diseases [23–28]. While few fMRI studies have been performed following RT to the brain, those that have report disruptions in functional network organization post RT [29, 30]. Our own case study of an adult patient who received a standard course of whole-brain RT without chemotherapy demonstrated substantial changes in functional network organization 8 months after completion of RT, including network-level changes corresponding to clinical outcome [31, 32]. In our work with pediatric brain tumor patients treated with proton beam radiation therapy, patients demonstrated significant alterations in brain-network organization, with profound changes in several association networks that subserve high-level cognitive functions[33].

Despite the promise of fMRI in assessing brain network disruption following RT, several factors complicate isolating the effect of whole-brain RT on brain network organization as it relates cognition. Compared to neural activity, blood-based measures of brain function are relatively slow and indirect. Cranial RT has known effects on the brain, including neuroinflammation [34], impaired neurogenesis [35], and vascular toxicity [36, 37]. Given the fact that tumors disrupt surrounding vasculature, any of these effects, either alone or in combination, could potentially complicate interpretation of blood-based brain assays [38]. Furthermore, RT-related changes in functional network organization have yet to be examined using animal models. A method that allows for mapping neural dynamics simultaneously with hemodynamics provides an opportunity to assess whether changes in network organization are a result of disrupted neuronal function and how systems-level measures of brain function correspond to cognitive dysfunction. To bridge these knowledge gaps, we developed a clinically-mimetic whole-brain RT protocol of 30 Gy in 10 fractions for use in mice. Longitudinal, awake wide-field optical imaging (WFOI) in Thy1-GCaMP6f mice tracked evolving cortical calcium and hemodynamic activity before and after whole-brain RT [39, 40], and extensive behavioral assessments measured RT-induced changes in cognitive performance.

## Material and methods

### Experimental design

**Fig. 1:**
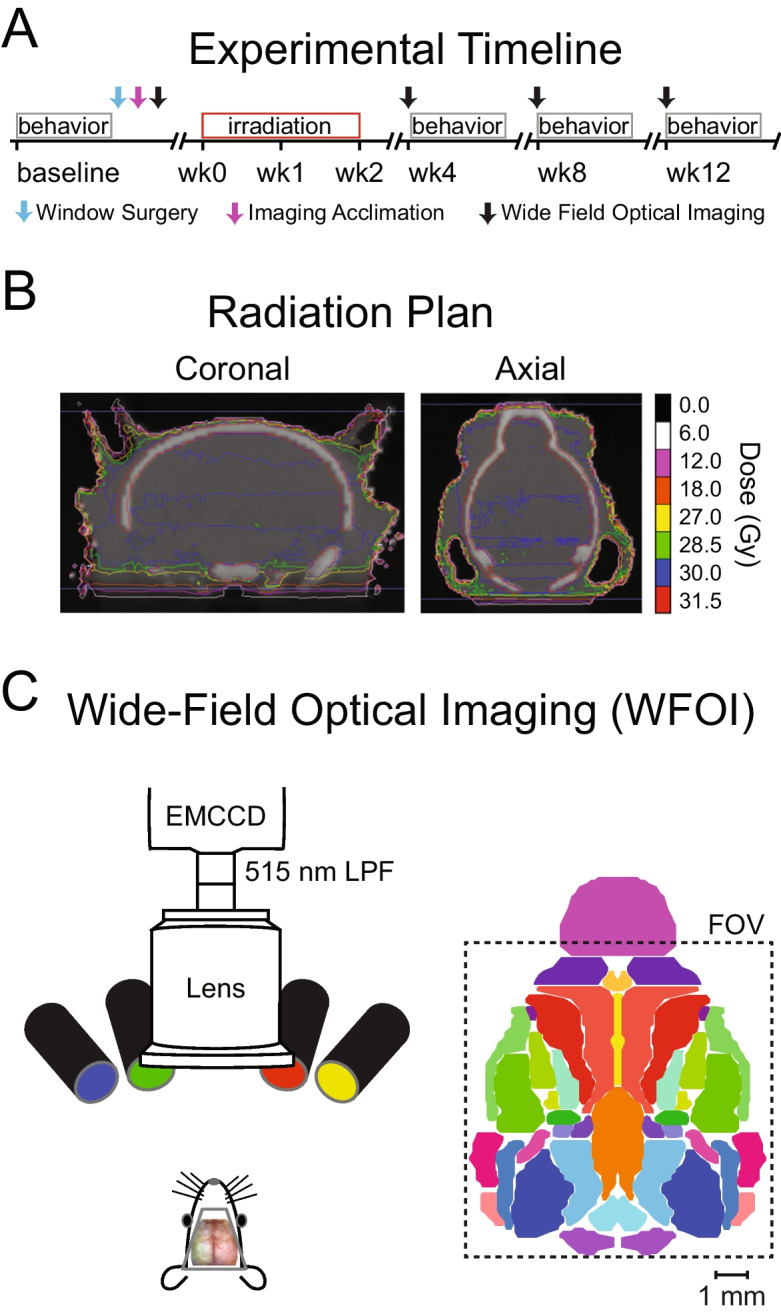
Experimental design, radiation plan, and WFOI system. (**A**) Experimental timeline: Baseline behavioral and imaging data were acquired prior to any RT (RT- Cohort; *N*=9). In a separate cohort (RT+ Cohort; *N*=17) mice received clinically mimetic fractionated whole-brain RT followed by behavioral testing and functional neuroimaging at 1 month, 2 months, and 3 months after whole-brain RT. (**B**) The whole-brain RT plan: A total of 30 Gy was delivered to each mouse in the RT+ Cohort via 10 fractions (3 Gy per day) over a period of 2 weeks. (**C**) Wide field optical imaging: A schematic of the imaging system is displayed to the left. The available field of view (approximately 1cm x 1cm) is displayed to the right (black box) on top of a dorsal view (same orientation as the schematic mouse on the left) of anatomical labels from the Paxinos Atlas for reference. RT=radiotherapy; Gy=Gray (Joule/kg)

Awake WFOI and behavioral testing occurred prior to fractionated RT (see Sections 2.2–2.4) in order to establish baseline metrics and then occurred monthly for 3 months after the full course of RT was completed (Fig. 1A). Behavioral testing consisted of a monthly set of sensorimotor and cognitive tests (see Section 2.6) to examine how RT-induced changes in brain activity and organization relate to behavioral performance. At the final time point, an additional cognitive test (fear conditioning) was administered. At each time point WFOI occurred 1 week prior to behavioral testing. Initial pilot studies revealed that RT compromised the optical quality of the cranial windows used for WFOI. Thus, experiments were performed in 2 cohorts. The first cohort (RT-) was prepared for WFOI, imaged, and then tested for behavioral performance. A second cohort (RT+) was subjected to fractionated RT over 2 weeks, prepared for WFOI, and then subjected to longitudinal WFOI and behavioral testing post RT for 3 months. All mice survived until the experimental endpoint, at which time they were sacrificed. The brain of each sacrificed animal was extracted and preserved for future histological analyses.

### Mice and animal preparation

All animal studies were approved by the Washington University School of Medicine Animal Studies Committee under guidelines and regulations consistent with the Guide for the Care and Use of Laboratory Animals, Public Health Service Policy on Humane Care and Use of Laboratory Animals, and the Animal Welfare Act and Animal Welfare Regulations. Animal reporting is performed according to ARRIVE guidelines.

A total of 26 transgenic Thy1-GCaMP6f mice were used in this study. Mice were originally acquired from Jackson Laboratories (JAX Strain: C57BL/6J-Tg(Thy1-GCaMP6f)GP5.5Dkim; stock: 024276) and bred in-house. Nine (5 female) mice (2–3 months old) were used for establishing baseline measures and 17 (9 female) mice (6–7 months old at the end of the experiment) were examined longitudinally following RT. Mice were housed in standard cages with ad libitum access to food and water in a dedicated animal facility under a 12-h light/12-h dark cycle.

Prior to awake WFOI, a small Plexiglas window was secured to the intact skull of each mouse with dental cement (Metabond) under isoflurane anesthesia following scalp retraction according to our previously published protocols [41, 42]. Briefly, mice were removed from their home cages and anesthetized with inhalation isoflurane (4.0% induction, 1.5% maintenance) and placed into a stereotaxic head fixation frame (Kopf Instruments, Tujunga, CA, USA). Each mouse was placed on a heating pad and temperature was maintained at 37°C via feedback from a rectal probe (mTCII Cell Microcontrols, Norfolk, VA, USA). The head was thoroughly shaved and cleaned, and a midline incision was performed. The scalp was retracted, and a custom-made clear Plexiglas window was affixed directly to the intact skull using dental cement (C & B Metabond, Parkell, Edgewood, NY, USA). Mice were monitored daily after window placement. Windows were pre-tapped with holes outside of the imaging field-of-view that were used to secure the head during imaging. Mice were behaviorally acclimated to the WFOI system over 1 week (once acclimated, they engaged in normal behavior such as grooming and whisking), allowing for continuous imaging over several hours.

### Whole-brain RT protocol

Our whole-brain RT protocol was designed to mimic those used for treating adult human brain metastasis in terms of its time course, fractionation scheme, and total amount of radiation delivered. The Small Animal Radiation Research Platform (SARRP) at Washington University was used to develop and deliver plans for whole-brain RT. The SARRP uses a 225kVp X-ray source, beam collimators, and a cone-beam computed tomography (CBCT) image-guided planning system. Mice were immobilized under isoflurane anesthesia for CBCT imaging, target definition, treatment planning, and irradiation. This process took <10min/mouse. A laser system allowed for visual confirmation of animal position and a CCD camera enabled the animal to be continuously monitored. The radiation plan was designed to deliver 30 Gy in 10 fractions (3.0 Gy/day at 3.5 Gy/min) to the whole brain using parallel-opposed lateral beams collimated to block the oral cavity and esophagus. Axial and coronal views of the plan are displayed in Fig. 1B.

### WFOI acquisition and preprocessing

WFOI was performed as previously described [39, 43]. A custom light engine consisting of four light emitting diodes (LEDs) centered at 470nm (LCS-0470-15-22 Mightex Systems, Pleasanton California, USA), 530nm, 590nm, and 625nm (M530L3-C1, M590L3-C1, M625L3-C1, Thorlabs, NJ, USA) illuminated the skull. The 470 nm LED was used for GCaMP6f excitation, while the other LEDs were used for multispectral oximetric imaging. Fluorescence emission and diffuse reflectance were collected by an 85 mm ƒ/1.4 camera lens (Rokinon, New York, New York, USA) attached to a cooled, frame-transfer EMCCD camera (iXon 897, Andor Technologies, Belfast, Northern Ireland, United Kingdom) with an approximately 1 sq cm field-of-view covering the majority of the dorsal cortex. A 515-nm long-pass filter (Semrock) was placed in front of the EMCCD to filter fluorescence excitation light and a 460/60 nm band-pass filter (Semrock) was used in front of the excitation source to minimize the contribution of fluorescence excitation light in the fluorescence emission channel (Fig. 1C). Images were acquired at a frame rate of 20Hz. The EMCCD acquired binned 4 × 4 pixels in order to increase SNR, giving a final resolution of 128 × 128 pixels (approximately 78 μm × 78 μm per pixel). Crossed linear polarizers in front of the LEDs and camera lens minimized specular reflection from the skull. The LEDs and EMCCD were synchronized and triggered via a data acquisition card (PCI-6733, National Instruments, Austin, TX, USA) using custom MATLAB scripts (MathWorks, Natick, MA, USA).

At each imaging time point 30 minutes of WFOI data were collected from each animal and stored in 5-min intervals. Prior to imaging, acclimated mice were placed in a felt pouch and their heads were secured via small screws in the Plexiglas windows. One second of dark images was collected prior to WFOI data collection, temporally averaged, and subtracted from all subsequent frames to remove dark counts from background noise sources. Each imaging session was analyzed for light level fluctuations (visual inspection of raw light-level traces, standard deviation per channel, and pixel offset over time) to identify and exclude any epochs exhibiting motion artifact. We subsequently excluded 230 min of resting state data (<13% of the total 1830 min collected across all mice).

All remaining pixel time traces were temporally detrended using a 5^th^ order polynomial fit to correct for any variations in light levels due to photobleaching, LED current drift, and other sources of nuisance signals. Reflectance changes were spectroscopically inverted to changes in hemoglobin concentration using the modified Beer-Lambert Law, as described previously [41, 44, 45]. Raw GCaMP6f fluorescence signals (∆F′) were corrected for changes in absorption due to hemoglobin using measured changes in hemoglobin concentration [46]. Images in each contrast were smoothed with a Gaussian filter (5 × 5 pixel box, *σ=*1.3). All data were temporally filtered between 0.01Hz and 5Hz and resampled to a frame rate of 10Hz to reduce data size. A binary brain mask was manually drawn in MATLAB for each recording session in each mouse. All subsequent analyses were performed on pixels labeled as brain. Image sequences from each mouse (as well as the brain mask for each mouse) were affine-transformed to Paxinos atlas space using the positions of bregma and lambda [47]. A group-level brain mask was created to limit analyses to the shared field-of-view of all mice. The resulting group-level brain mask included 9728 out of 16384 possible pixels. For each mouse the global signal from within the shared brain mask was regressed from all data, following standard human fMRI preprocessing algorithms [48].

### Imaging data analyses

#### Power analysis

The power spectral density (PSD) of each time trace was calculated using the pwelch function in MATLAB. Each trace was divided into 8 segments with 50% overlap and windowed by a Hamming function. The periodogram of each segment was calculated via the discrete Fourier transform, squared, and all 8 periodograms were averaged. PSD curves for each mouse were calculated by averaging PSD curves across all brain pixels and runs. For each mouse, power spectral density was computed at each pixel across the full spectrum (0.01–5 Hz) and separately for either the delta band (calcium data only; 0.5–4.0 Hz) or the infraslow band (hemoglobin data only; 0.01–0.1 Hz). Full spectrum data were averaged across all pixels in the field of view for each mouse.

#### Functional connectivity analyses

Functional connectivity (FC) was evaluated via zero-lag correlation across all pairwise comparisons in the group-level brain mask, as per our prior reports [49]. For each mouse, FC matrices were calculated for each valid run and averaged together, resulting in one 9728x9728 element FC matrix per mouse. Within each cohort of mice and at each experimental time point (RT-, RT+1mo, RT+2mo, and RT+3mo) group-averaged FC matrices were computed by averaging together all of the individual matrices for each cohort and/or time point. All individual matrices were Fisher-Z transformed before averaging. Average matrices were inverse Fisher-Z transformed for display in figures.

The abovementioned whole-cortex FC matrices contain ~47 million nonredundant correlation coefficients, many of which are not independent (e.g., due to spatial autocorrelation). In order to compress this information, each whole-cortex FC matrix was parcellated into a set of functional networks. Thus, all whole-cortex FC matrices (one for each mouse at each experimental time point and one for each group) were sorted via the final InfoMap consensus solution (see Section 2.5.3.), ensuring that sets of pixels that are in the same network community were grouped together. Functional networks are delineated by horizontal and vertical black lines overlaid onto the pixel-wise FC matrices. Within each of these “network blocks” (i.e., the set of pixels within a box created by the black lines), pixels were sorted anatomically from anterior-to-posterior, left-to-right. For further analyses, all non-diagonal correlation coefficients within a given block were averaged together to create a network-averaged FC matrix resulting in a 9×9 element FC matrix. This procedure both normalizes each functional network by size and acts a data reduction technique for statistical analyses. Differences in FC were computed for the RT- and RT+ Cohorts versus one another (via matrix subtraction) at each experimental time point using the group-average, block-average FC matrices.

#### InfoMap

Group-average functional network organization was determined for the RT- cohort using InfoMap [50]. InfoMap is a deterministic, data-driven community detection algorithm that has been widely used in human fMRI studies in order to identify functional networks of the brain [51]. Briefly, InfoMap deploys a random walker to explore the network (FC matrix) in graph space, where the link or edge between any pair of network nodes represents the correlation coefficient between those nodes (here a node represents one pixel in the group-level brain mask). The random walker transitions from one node to another with a probability directly equal to the weight (i.e., the correlation coefficient) of the edge/link connecting those nodes. The algorithm optimizes an objective function using information theory, with the goal of minimizing the amount of information needed to label network communities with zip codes.

Here, InfoMap was applied to the unsorted, group-average, pixel-wise FC matrix after it was thresholded down to a specified edge density (i.e., to include only the strongest X% of correlation coefficients), since InfoMap, and most other community detection algorithms, requires a sparse matrix. It was run using 10,000 iterations (the random walker’s starting position changes on each iteration) for each edge density tested (5, 6, 7, 8, 9, 10, 15, 20, 25, and 30%). An exclusion distance of 5 pixels (~390 μm) was applied to account for spatial autocorrelation, meaning that when the matrix is thresholded, any nodes within 5 pixels or fewer from one another (in brain space) are not allowed to have an edge between them. After completing all iterations, InfoMap outputs one solution for each edge density threshold in the form of a vector of network labels (one for each node/pixel). After running InfoMap for each edge density, a pixels-by-thresholds (9728 x 10) matrix of network labels was created. Network labels were matched across thresholds so that “network 1” is the same network for each threshold.

While each solution for a given edge density threshold is deterministic (given enough iterations), solutions across edge densities do not necessarily need to agree, e.g., a sparser threshold will result in more functional network communities identified relative to a denser threshold. In order to synthesize solutions across the range of edge densities tested, a consensus was formed following the procedure of Laumann, et al. [52]. The consensus network label for each node was assigned at the sparsest possible threshold at which that node successfully received a label (for all nodes that InfoMap cannot identify as belonging to a distinct network community it assigns those nodes as “unlabeled”). The final consensus solution included 9 functional networks: Parietal, Somatomotor, Retrosplenial, Posterior Retrosplenial, Anterior Cingulate, Secondary Visual, Primary Visual, Frontal, and Auditory (this is the order by which each FC matrix is sorted).

#### Network segregation analysis

Network segregation was computed separately for sensorimotor and association systems for each experimental time point following the procedure of previous reports [53]. Whole-cortex pixel-wise FC matrices were used for this analysis. Network segregation measures the balance of within- and between-network correlations for a given set of networks. Here, sensorimotor systems were defined as the Somatomotor, Visual (both), and Auditory networks, while association systems were defined as the Parietal, Frontal, Cingulate, and Retrosplenial (both) networks. A key difference between the computation of network segregation performed by Chan and colleagues and the computation performed here is the inclusion of negative correlation coefficients, which allows for segregation values to exceed 1. Negatives were included to be consistent with the methods used for the results presented in Fig. 4 from human pediatric brain tumor patients [33].

### Behavioral testing and analysis

Behavior tests were conducted in the Washington University Animal Behavior Core. Mice were acclimated to the behavioral testing room for 1 week prior to evaluation. Mice were tested during the light phase of the light/dark cycle by a female experimenter blind to treatment condition. Three separate cohorts of C57BL/6J mice were used for phenotypical analysis (*N* = 19 RT and *N* = 14 control). Each cohort was tested using a one-hour locomotor activity test, a sensorimotor battery, a novel object location test, and a startle response and pre-pulse inhibition test. Mice were tested at baseline and then monthly for 3 months after completion of RT. A conditioned fear test was administered only at the 3-month time point following completion of the other tests.

One-hour locomotor activity. To evaluate general activity levels and possible alterations in emotionality, mice were evaluated over a 1-h period in transparent (47.6 × 25.4 × 20.6 cm high) polystyrene enclosures. Each cage was surrounded by a frame containing a 4 × 8 matrix of photocell pairs, the output of which was fed to an on-line computer (Hamilton-Kinder, LLC, Poway, CA). The system software (Hamilton-Kinder, LLC) was used to define a 33 × 11 cm central zone and a peripheral or surrounding zone that was 5.5 cm wide with the sides of the cage being the outermost boundary. Variables that were analyzed included the total number of ambulations and rearing on hindlimbs, as well as the number of entries, time spent, and distance traveled in the center area as well as the distance traveled in the periphery.

Sensorimotor battery. Walking initiation, ledge, platform, pole, and inclined and inverted screen tests were performed to assess sensorimotor function. Time in each task was manually recorded. The average for two trials was used for analyses. Test duration was 60s, except for the pole test, which was extended to 120s. For walking initiation, time for an animal to leave a 21×21cm square on a flat surface was recorded. For ledge and platform tests, the time the animal was able to balance on an acrylic ledge (0.75cm wide and 30cm high), and on a wooden platform (1.0cm thick, 3.0cm in diameter and elevated 47cm) was recorded, respectively. The pole test was used to evaluate fine motor coordination by quantifying time to turn 180° and climb down a vertical pole. The screen tests assessed a combination of coordination and strength by quantifying time to climb up or hang onto a mesh wire grid measuring 16 squares per 10cm, elevated 47cm and inclined (60° or 90°) or inverted.

#### Novel object location recognition

Recognition memory was evaluated using a novel object location task. Each mouse was habituated to the test chamber over 2 days and on the third day given a sample trial and a test trial. For novel object location, during the sample trial mice are placed in the familiar arena with two copies of the same object placed in opposite corners of the arena. Mice are allowed to explore for 10 minutes, and then returned to their home cage for a 50-min delay, then placed back into the test arena where one of the objects has been moved to a novel location. The amount of time each animal spends actively investigating the objects is scored using ANY-Maze tracking software (Stoelting, Inc). Investigation zones are set within a 2cm perimeter of each object, and investigation is counted if the animal’s nose is located within the investigation zone.

#### Acoustic startle response (ASR) and pre-pulse inhibition (PPI)

In the first cohort, the ASR to a 120 dB auditory stimulus pulse (40 ms broadband burst) and PPI (response to a pre-pulse plus the startle pulse) were measured concurrently in the mice. Beginning at stimulus onset, 1 ms force readings were averaged to obtain an animal's startle amplitude. A total of 20 startle trials were presented over a 20 min test period during which the first 5 min served as an acclimation period when no stimuli above the 70 dB white noise background were presented. The session began and ended by presenting 5 consecutive startle (120 db pulse alone) trials unaccompanied by other trial types. The middle 10 startle trials were interspersed with PPI trials, consisting of an additional 30 presentations of 120 dB startle stimuli preceded by pre-pulse stimuli of either 4, 12, or 20 dB above background (10 trials for each PPI trial type). A percent PPI score for each trial was calculated using the following equation: $\% PPI=100\times \frac{ASR_{startle\ pulse\ alone}-\left({ASR}_{prepulse+ startle\ pulse}\right)}{ASR_{startle\ pulse\ alone}}$. The third cohort was tested using an upgraded version of the previously used Kinder Scientific Startle/PPI equipment. A paired *t*-test comparing 10 startle trials and 10 non startle trials for each mouse was used to identify deaf mice. All mice included in the study passed the deaf test.

Conditioned fear. Two distinct chambers were used to train and test mice (26×18×18 cm high) (Med-Associates, St. Albans, VT), which were easily distinguished by different olfactory, visual, and tactile cues present in each chamber. On day 1, each mouse was placed into the conditioning chamber for 5 min and freezing behavior was quantified during a 2 min baseline period. Freezing (no movement except that associated with respiration) was quantified using FreezeFrame image analysis software (Actimetrics, Evanston, IL) which allows for simultaneous visualization of behavior while adjusting for a “freezing threshold” during 0.75 s intervals. After baseline measurements, a conditioned stimulus (CS) consisting of an 80 dB tone (white noise) was presented for 20 s followed by an unconditioned stimulus (US) consisting of a 1 s, 1.0 mA continuous foot shock. This tone-shock (T/S) pairing was repeated each minute over the next 2 min, and freezing was quantified after each of the three tone-shock pairings. Twenty-four hours after training, each mouse was placed back into the original conditioning chamber to test for fear conditioning to the contextual cues in the chamber. This involved quantifying freezing over an 8 min period without the tone or shock being present. Twenty-four hours later, the mice were evaluated on the auditory cue component of the conditioned fear procedure, which included placing each mouse into the other chamber containing distinctly different cues. Freezing was quantified during a 2 min “altered context” baseline period as well as over a subsequent 8 min period during which the auditory cue (CS) was presented. Shock sensitivity was evaluated following completion of the conditioned fear test.

### Statistics

All statistical analyses for imaging measures were performed using the MATLAB Statistics and Machine Learning Toolbox (R2018b). Changes in cortical power over time were evaluated using analysis of variance (ANOVA) and all significant main effects of time were evaluated using post hoc *t*-tests. FC changes within the RT+ Cohort were assessed via repeated measures ANOVA using mouse-level block-average FC matrices. FC changes across time (RT- and RT+ Cohorts) were assessed via one-way ANOVA. Separate ANOVAs were computed for each network block, and these results were corrected for multiple comparisons using False Discovery Rate (FDR) correction. Specific within-network FC blocks that survived multiple comparison correction were tested against the RT- Cohort at each experimental time point using two-sample *t*-tests. These results were also FDR-corrected. Network segregation results were tested for significance using either two-sample (RT- vs. RT+) or paired (RT+ vs. RT+) t-tests. Correlational relationships were explored between within-network FC and non-sensorimotor behavioral measures. In order to limit multiple comparisons, only measures that exhibited significant changes after whole-brain RT were included. Pearson correlations were computed between each FC variable and behavioral measure. The alpha level for significance was set to 0.05 before FDR correction.

All statistical analyses for the behavioral measures were performed using IBM SPSS Statistic software (v.26) and GraphPad Prism (v.9). ANOVAs (one-way, factorial, repeated-measures) were used analyze the behavioral data. Analysis of covariance (ANCOVA) was used to analyze conditioned fear data to control for differences in baseline activity levels. With a statistically significant interaction between main factors, simple main effects were calculated to provide clarification of statistically significant between-genotype and within-genotype differences. Where appropriate, the Huynh-Feldt adjustment was used to protect against violations of sphericity, the Bonferroni correction was applied to multiple pairwise comparisons and post hoc tests. Probability value for all analyses was *p* < .05, unless otherwise stated.

## Results

### Widespread changes in brain function and network architecture following fractionated whole-brain RT

**Fig. 2:**
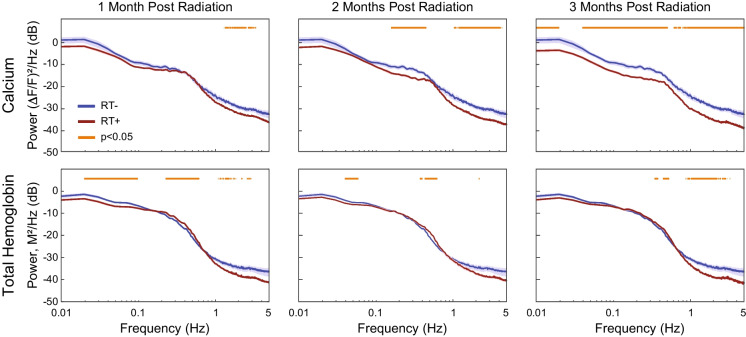
Spectral changes after whole-brain RT. Clinically mimetic whole-brain RT significantly disrupted brain function in mice. Both calcium (top row) and oxygen (bottom row) signaling were affected. Global changes in calcium and total hemoglobin power spectral density were observed at all time points after whole-brain RT (red lines) relative to the RT- Cohort (blue lines). Light red horizontal lines at the top of each plot indicate the frequencies that passed FDR-correction. Neural signaling (as measured by calcium) was significantly suppressed in the delta band (0.5-4 Hz) 1-month after whole-brain RT (left column), and this suppression increased in magnitude at both 2-months (middle column) and 3-months (right column) after whole-brain RT. Moreover, calcium power significantly decreased over time across nearly the entire measured spectrum in the RT+ Cohort, as assessed via repeated-measures ANOVA, resulting in significant decreases at most frequencies (relative to the RT- Cohort) at the 3-month time point. Significant decreases in infraslow hemodynamic activity (similar to BOLD-FMRI fluctuations) were more modest 1-month after whole-brain RT and recovered by the 3-month time point. Hemodynamic changes higher than approximately 1 Hz represent changes in physiological signaling (heart rate, respiration) and were not evaluated further

Previous fMRI studies in adult patients treated with whole-brain RT have observed widespread changes in hemodynamic activity and functional network architecture [54, 55]. However, whether these changes correspond to underlying neural signaling and function has not been demonstrated. WFOI in Thy1-GCaMP6f mice allowed for simultaneously measuring hemodynamic and neural signaling before and after delivery of whole-brain RT. Relative to unirradiated (RT-) mice, RT significantly reduced global cortical neuronal activity over time (F statistic ranged from 18 to 35, *p*<0.0001 via rmANOVA for all frequencies) (Fig. 2, top Row). For example, at 1 month following RT (RT+1mo) activity between 1.3 and 3.5 Hz (i.e., within the delta band) was significantly reduced by an average of 42% compared to controls. This activity remained suppressed 2 months after RT (average of 56% reduction from 1-4 Hz), with slower frequencies (approximately 0.1–0.5 Hz) also exhibiting an average 47% decline in power. Three months post RT (RT+3mo) we observed significant broadband reductions in excitatory activity globally over the cortex (66% reduction averaged over all significantly affected frequency bins). At the experimental end point, delta band calcium activity in mice receiving whole-brain RT was 67% less than RT- mice. A different picture was observed when examining hemodynamic activity (Fig. 2 bottom row). Relative to RT- mice, modest broadband decreases (23% of controls, from approximately 0.02–2.3 Hz) in total hemoglobin were observed 1 month following RT. Further, these changes were inconsistent with respect to frequency range or time point. For example, over infraslow frequencies (0.01–0.1 Hz, analogous to ranges used for human fMRI studies), there was a significant 32% decrease in power 1 month after whole-brain RT that was no longer significant at the 3-month time point (*p*>0.05). Hemodynamic changes higher than ~1 Hz represent changes in physiological signaling (e.g., heart rate, respiration) and are not related to changes in spontaneous calcium activity associated with brain network organization. Our results suggest that whole-brain RT initially affects faster (i.e., delta band) excitatory signaling followed by progressive reductions in neuronal activity over wider and slower frequency ranges as time continues after RT. Concomitant changes in hemodynamic activity were not observed following RT, suggesting that whole-brain RT differentially affects vascular and neuronal signaling.

**Fig. 3:**
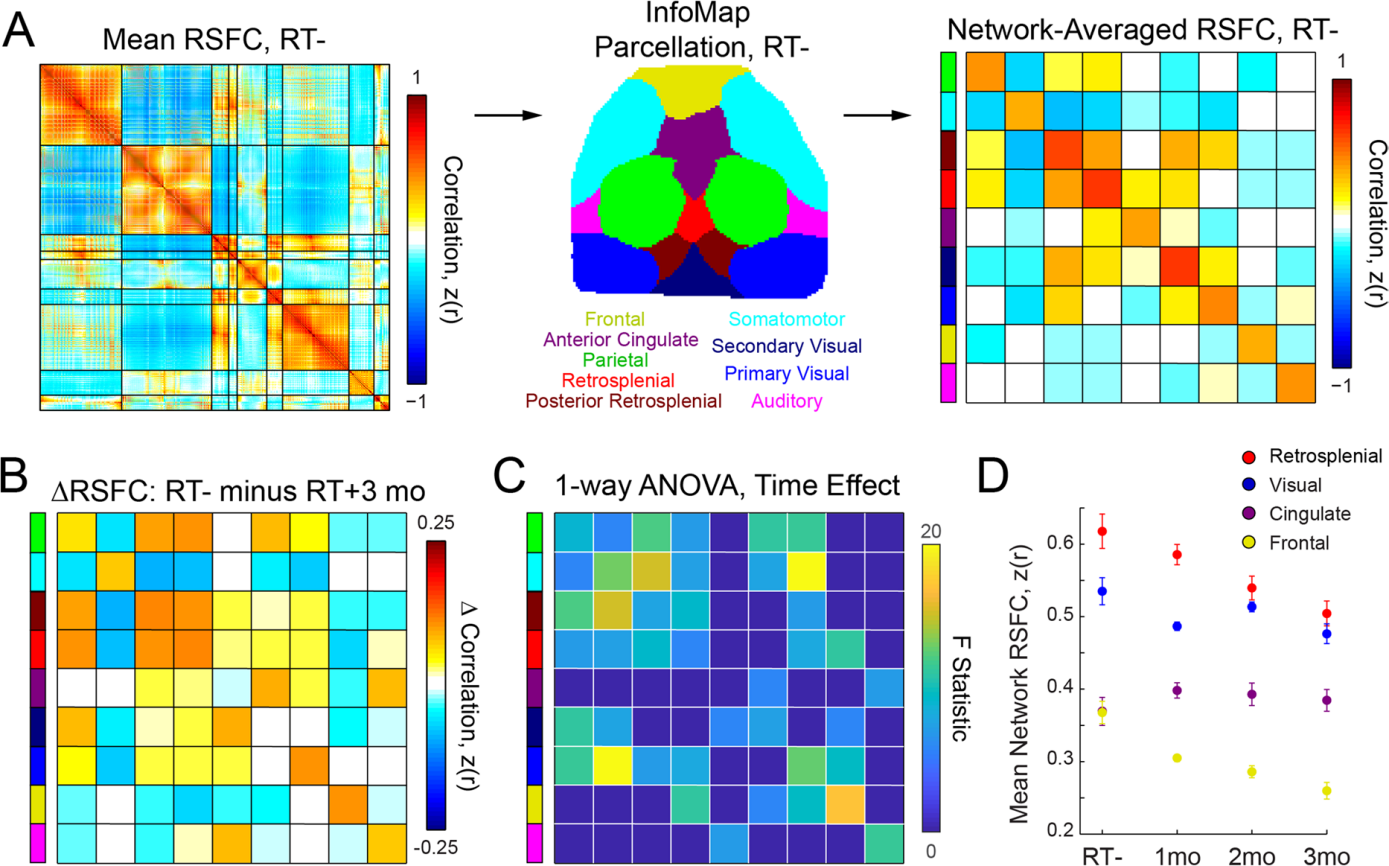
Brain network architecture is significantly disrupted after whole-brain RT. Clinically mimetic whole-brain RT significantly disrupted brain organization in mice. (**A**) The group-average FC matrix for the RT- Cohort is displayed. Each entry in the matrix represents the mean (across mice) correlation coefficient between delta-band calcium signals for a given pair of pixels. Functional networks are delineated by horizontal and vertical black lines. Functional network organization was determined via the InfoMap consensus solution (see Section 2.5.3), which is displayed in the middle. The matrix on the right displays block-average FC for the RT- Cohort (see Section 2.5.2). Each network block is labeled with a colored bar on the y-axis, where the colors denote the specific functional network to which each block corresponds. (**B**) The difference between block-average FC for the RT- and RT+3mo Cohorts is displayed. Each entry in the matrix corresponds to the change in FC for the network block, with warmer colors indicating that FC was more positive for the RT- Cohort and colder colors indicating that FC was more positive for the RT+3mo Cohort. (**C**) The matrix displays the F-statistic from a one-way ANOVA (RT- and RT+ Cohorts) computed across all time points. A separate ANOVA was computed for each network block. All non-zero blocks passed False Discovery Rate (FDR) correction. There was a significant main effect of time in every network except the Anterior Cingulate (purple). (**D**) A repeated-measures ANOVA (RT+ Cohort only) revealed significantly diminished within-network block-average FC over time for the Frontal (yellow) and Retrosplenial (red) networks, but not for the Anterior Cingulate (purple) and Visual (blue) networks. Within-network FC for the RT- Cohort is also displayed for reference. Error bars correspond to standard error from the mean (across mice). FC=functional connectivity

Higher order functional networks appear to change most following whole-brain RT in human studies, with both the dorsal attention network [55] and frontoparietal control networks [54, 55] exhibiting the largest changes. To examine the impact of whole-brain RT on neuronal network architecture in mice, we used group-average whole cortex FC in RT- mice (Fig. 3A left) to identify functional systems. To do so, we used a deterministic, data-driven community detection algorithm (InfoMap; see Section 2.5.3.) that yielded 9 networks (Fig. 3A middle): Parietal (green), Sensorimotor (light blue), Frontal (yellow), Posterior Retrosplenial (dark red), Retrosplenial (red), Anterior Cingulate (purple), Secondary Visual (dark blue), Primary Visual (blue), and Auditory (pink). Block-average FC matrices (average within each network block after sorting the matrices; see Section 2.5.2.) were computed for each mouse across all time points (Fig. 3A right). Whole-brain RT significantly impacted global brain network organization (Fig. 3B, only RT+3mo shown for simplicity). Significant main effects of time were observed when examining within-network changes (Fig. 3C main diagonal; *F*=5.8 to 17.2, *p*=0.0015 to <0.0001) for all systems except the Anterior Cingulate (purple; *F*=0.5, *p*=0.65). Further, significant between-network interaction effects (Fig. 3C off-diagonals; *F*=5.7 to 20.0, *p*=0.0019 to <0.0001) were broadly observed across all systems. Post-hoc analysis revealed significant decreases in FC strength (relative to RT-) occurred only within the Parietal (*t*=4.1, *p*=0.0005) and Frontal (Fig. 3D; *t*=4.9, *p*=0.0001) networks 1-month after whole-brain RT. Decreases in Frontal, Posterior Retrosplenial, Primary Visual, and Parietal within-network FC were significant 2-months after whole-brain RT (*t*=2.9 to 5.1, *p*=0.0078 to <0.0001), and in Frontal, Parietal, Somatomotor, Primary Visual, Retrosplenial, Posterior Retrosplenial, and Auditory (*t*=3.4 to 5.6, *p*=0.002 to <0.0001) 3-months after whole-brain RT. The Anterior Cingulate network was unaffected over time (Fig. 3D purple).

**Fig. 4:**
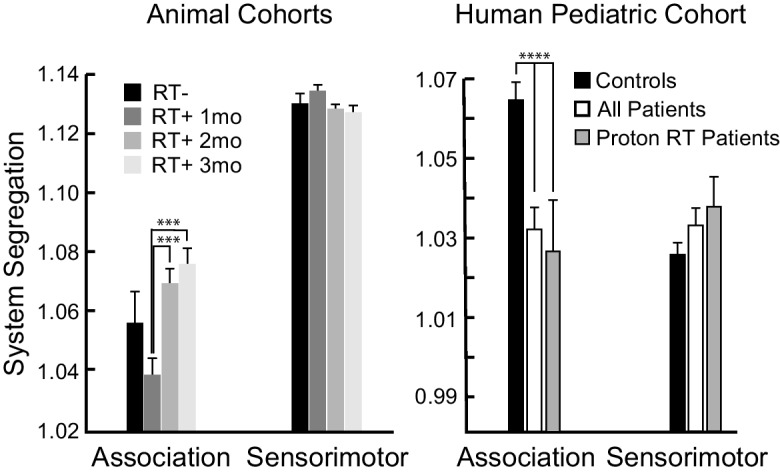
Functional network segregation may be a translational measure. Converging evidence suggests that segregation between functional systems is an important feature of brain network organization. Association systems, i.e., those that are found in association cortex, are thought to subserve high-level cognitive functions in humans. Prior work has found that association system segregation declines over several decades in healthy older adults and that more rapid declines are related to long-term impairments in cognitive function. Previous work from our group revealed an acute decrease in association system segregation in pediatric brain tumor patients (right graph, white bar; ~3 years after diagnosis, on average), but no change in sensorimotor system segregation compared to controls (black bar). These effects were consistent when analyzing only patients who received proton beam RT (gray bar). Here, we observed a non-significant acute decrease in association system segregation 1-month after whole-brain RT that then significantly increased over time. This segregation recovery phenotype was not present in the sensorimotor systems, whose segregation remained constant over time, similar to human patients. Association system segregation may prove to be a translational and transdiagnostic biomarker of cognitive injury

Whole-brain RT differentially affected network FC strength. We quantified this effect using a repeated-measures ANOVA (data from the RT+ Cohort only). For the Frontal and Retrosplenial networks there were significant decreases in FC over time (Fig. 3D yellow and red; *F*=6.6 to 15.8, *p*=0.004 to <0.0001). Likewise, there was a significant increase (*F*=7.8, *p*=0.0019) in FC over time for the between-network interaction corresponding to the Parietal and Somatomotor networks. This increase in between-network FC was due to the loss of anti-correlation between the Parietal and Somatomotor networks after whole-brain RT. These three exemplars highlight a general trend in loss of FC magnitude, whether positive or negative, after whole-brain RT. Such widespread alterations in FC (moving towards zero, see Figure S1) contributed to diminished network segregation (Fig. 4A; see Section 2.5.4.). However, changes in network segregation were not significant (*p*=0.29 to 0.57) in systems relating to sensorimotor functions (motor control, vision, audition, and somatosensation). There was a recovery phenotype in association system segregation after whole-brain RT, with an initial reduction (not significant; *t*=1.6, *p*=0.1) and then significant increase over time (*t*=4.0 and 4.7, *p*=0.0003 and <0.0001). Figure 4 also includes a comparison of this network segregation result with a similar result from human pediatric brain tumor patients [33], in which association system segregation (and only association system segregation) is significantly decreased ~3 years after initial diagnosis. This result is consistent when limiting analysis to patients who only received proton beam RT [33]. Therefore, our evidence suggests that clinically-mimetic whole-brain RT causally disrupts neuronal signaling and whole-brain functional network organization.

### Clinically mimetic whole-brain RT impairs sensorimotor and cognitive performance

To determine whether RT-induced changes in brain organization were associated with behavioral performance, mice were subject to a monthly set of sensorimotor and cognitive tests. Relative to the RT- group, a significant main effect of whole-brain RT (*p* = 0.009) and sex (*p* = 0.009), as well as a significant RT x sex interaction effect (p = 0.003) was observed on locomotor activity (Figure S2A) with RT+ mice generally exhibiting decreased locomotor activity. Likewise, there was a main effect of sex (p = 0.039) and a significant whole-brain RT x sex interaction effect (p = 0.032) on exploratory rearing behavior relative to the RT- cohort (Figure S2B). The largest decreases in exploration were observed in RT+ female mice; however, there was no main effect of whole-brain RT on rearing. There was also a main effect of whole-brain RT (*p* = 0.006) on center zone exploration, resulting in decreased exploratory behavior relative to the RT- cohort (Figure S2C). No significant differences in 90-degree screen climbing time were observed following whole-brain RT (Figure S2D).

**Fig. 5:**
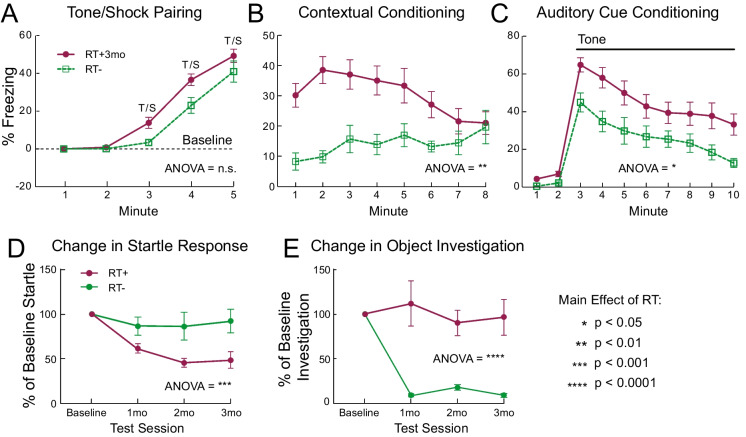
Effects of whole-brain RT on high-level behavioral functions. The results from each non-sensorimotor behavioral test are displayed, except for the pre-pulse inhibition test, which did not result in significant differences after whole-brain RT. (**A**) There was no difference in responses to the tone/shock pairing during the conditioned fear test after accounting for differences in general activity levels between RT- (green lines/dots) and RT+ (purple lines/dots) mice. However, there were significant increases in anxiety-like behaviors (increased freezing) in the RT+ mice after fear conditioning during subsequent tests involving the learned (**B**) contextual/environmental and (**C**) auditory cues. Furthermore, whole-brain RT caused significant longitudinal deficits in (**D**) startle responses to an auditory stimulus and (**E**) recognition memory (novel object location test). All error bars represent standard error from the mean

During the conditioned fear test, there were no significant differences in freezing behavior between cohorts at baseline nor after the tone-shock presentations after controlling for differences in general activity levels (Fig. 5A). However, we observed a significant main effect of whole-brain RT, even after controlling for differences in activity levels, on freezing behavior in response to contextual cues (Fig. 5B; *p* = 0.009) and auditory cues (Fig. 5C; *p* = 0.042) alone. Further, there was a significant main effect of sex (p = 0.019) and significant sex x RT interaction effect (*p* = 0.04) on auditory cues. Prior to any tests involving an auditory component, all mice were evaluated to ensure significant responses to auditory stimuli. Startle responses to auditory stimulus resulted in a significant main effect of whole-brain RT (*p* < 0.001) (Fig. 5D). No significant differences were observed in in pre-pulse inhibition of the startle response (Figure S2E). As part of the cognitive set of behavioral tests, we observed a significant main effect of whole-brain RT (*p* < 0.0001) on novel object investigation (Fig. 5E). Mice in the RT+ cohort showed significantly higher interest in the novel objects upon repeated testing, suggesting impaired habituation. This impairment was not observed in RT- mice. Notably, there was no significant difference in general movement during the novel object test (Figure S2F), implying that the observed effects cannot be explained by differences in activity levels. Together, our evidence suggests that clinically-mimetic whole-brain RT causally disrupts behavioral performance, including high-level (non-sensorimotor) cognitive functions.

Our ultimate goal for developing a clinically-relevant animal model of whole-brain RT that is isolated from confounds such as chemotherapy is to understand systems-level brain effects that predict cognitive decline, thereby leading to potential clinical targets for intervention. In order to work towards this goal, we must first understand the complicated, multiplexed effects of whole-brain RT on brain network organization, behavior, and the relationship between them. Here, we examined correlations between significant changes in within-network FC strength and significantly affected non-sensorimotor behaviors, i.e., contextual cue conditioning, auditory cue conditioning, and novel object investigation. There were no significant relationships between any network and any behavioral measure.

## Discussion

We developed a clinically-relevant mouse model of whole-brain radiotherapy (RT) and analyzed its effects on systems-level brain organization, neuronal function, and behavior. RT planning was performed to deliver the same dose and fractionation as received by human patients with brain metastases (30 Gy in 3 Gy/fraction over 2 weeks). All mice survived until the experimental end point, indicating a robust and reliable whole-brain RT protocol. The effects on brain function and organization were evaluated using longitudinal wide-field optical imaging (WFOI) in awake animals to monitor evolving cortical calcium activity and hemodynamics following RT. A wide variety of behavioral domains were tested for evaluating the effects of whole-brain RT on sensorimotor and cognitive functions.

Whole-brain RT remains an important treatment approach for patients with cancer. In adults, it is largely used for the treatment of brain metastases when radiosurgery is impossible due to the size and number of tumors. With advances in systemic therapy, more patients receiving whole-brain RT are living to become long-term survivors with increased risk for radiation-associated encephalopathy and dementia [56]. Pre-clinical studies of whole-brain RT in mice allow for more direct investigation of the effects of radiation on the brain. There is a robust literature about radiation-induced cognitive dysfunction in mice. RT is associated with neurogenesis inhibition in the hippocampus, astrocyte proliferation, increased blood brain permeability, and vascular changes [57–60]. It has been shown that these affected tissues are vulnerable to radiation with a dependence on dose per fraction, total dose, and dose rate [61, 62]. This is attributable to the low α/β ratio of neural tissues, meaning that these tissues have greater ability to repair between fractions and have high sensitivity to large fraction size [56, 63, 64]. For these reasons, it is important for animal studies evaluating neural changes to RT to follow fractionation and dose delivery schedules that are reflective of what is utilized in humans [65, 66]. While whole-brain RT dose and fractionation schedules studied in humans have ranged from 10 Gy in a single fraction [67, 68] or 12 Gy in 2 fractions [69] to 30 Gy in 10 fractions [67, 68, 70–73] or 50 Gy in 20 fractions [70, 74], by far the most commonly utilized regimen is 30 Gy in 10 fractions, which was chosen for the regimen in this study [71].

Using WFOI, we observed that whole-brain RT decreases global excitatory signaling over the cortex. However, the effects of whole-brain RT preferentially affected faster (delta band) activity at earlier time points (1 month post RT) compared to progressive, broadband decreases that included intermediate (0.1–1 Hz) and then infraslow frequency ranges (0.01–0.1 Hz) at later time points (3 months post RT). Radiation damage to normal tissues has historically been attributed to DNA damage and the loss of proliferative cells. However, recent evidence revealed that RT results in synaptic dysfunction in surviving neurons [75], and plays a role in cognitive dysfunction following RT. Our observations of progressive decline in excitatory signaling is also consistent with other work examining short- and long-term effects of radiation exposure. For example, acute neuronal hypofunction attends long-lasting brain dysfunction due to diminished synaptic excitatory signaling at NMDA receptors, and long-term changes in the composition of the post-synaptic density [76]. Thus, early radiation-induced abnormalities in neuronal signaling may lead to persistent functional disruption in synaptic communication [77]. It is important to note that genetically encoded calcium indicators do not completely capture neuronal activity [78, 79]. Furthermore, reductions in excitatory activity observed following RT could be due to increased activity in subpopulations of inhibitory cells [45]. For example, radiation leads to increased surface expression of gamma-aminobutyric acid receptors (GABAARs) and elevated GABAAR-mediated responses in slice preparations, yet in vivo radiation is associated with increased GABA release from inhibitory basket cells onto pyramidal neurons [75]. Future studies can examine the correspondence between early changes in inhibitory/excitatory signaling and long-term cognitive outcome as a potential therapeutic target.

Coherent patterns of spontaneous activity within and across hemispheres represent highly organized synaptic signaling throughout the brain [80]. Through neurovascular coupling, patterns of spontaneous (and task-driven) neural activity manifest as fluctuations in blood oxygenation (e.g., via BOLD-fMRI). These spontaneous patterns of activity are used to define widely-distributed topographies known as resting-state networks (RSNs) that correspond to known sensory, motor, and higher-order functional systems, such as those thought to subserve cognitive functions [21, 81, 82]. Mapping resting-state functional connectivity (RSFC) within and across RSNs has proven to be a sensitive tool for evaluating brain integrity [21, 22]. For example, RSFC strength can be modified by learning [83–85], is predictive of future performance on behavioral tasks [86–88], and is an important biomarker of functional and behavioral recovery after injury [24–27, 89–93]. However, data acquired with fMRI is often used as a surrogate of underlying neuronal activity, despite these processes not being directly measured. These efforts are critical to interpreting human fMRI data, since one may not be able use the BOLD signal to infer/measure bulk neural activity in the context of atypical neurovascular functioning. Here, we take advantage of genetic engineering approaches in mice to visualize how fractionated irradiation affects fluctuations in calcium concentration in primarily excitatory cells [94].

We and others [39, 46, 95–97] have reported coupling between neuronal calcium transients and subsequent hemodynamics, each of which can inform underlying functional network architecture demonstrated by patterns of RSFC. In the present study, functional networks were identified in healthy (RT-) adult mice using a data-driven community detection technique (InfoMap) that is widely used in human fMRI studies. Applying representations of ‘typical’ murine functional networks to mice receiving whole-brain RT revealed RSFC disruption at 1 month post RT that persisted and worsened over 3 months. Specifically, both within- and between-network RSFC was generally suppressed (i.e., moved towards zero). The largest changes (reduced FC strength) were observed within the Retrosplenial and Frontal networks. Further, there was a trend towards decreased network segregation at 1-month post RT in non-sensorimotor regions. Sensorimotor network segregation was unaffected by whole-brain RT. All of the abovementioned results converge with fMRI studies of human brain tumor patients, which report that the largest changes occur in so-called association systems (i.e., the set of functional networks in association cortex) [33]. Association system segregation, but not sensorimotor system segregation, is significantly decreased in pediatric brain tumor patients treated with proton beam radiation therapy [33], and FC strength was most affected in the Medial Temporal Lobe and Frontoparietal networks in an adult patient who received whole-brain RT without chemotherapy [54]. However, the present work isolates the effects of whole-brain RT alone and avoids the confounds of solid tumor presence in the brain, surgery, chemotherapy, hormone therapy, and all other standard methods of care used in human patients.

In addition to network-level changes, we observed significant alterations in behavioral performance. Non-sensorimotor changes included decreased recognition memory and increased anxiety-like behaviors. In humans, functional networks in retrosplenial, medial temporal, and hippocampal cortex, as well as networks spanning large portions of dorsolateral prefrontal cortex are thought to subserve such behaviors [98, 99]. Thus, both behavioral and functional network changes are broadly consistent with one another in human patients. We observed analogous consistencies in the mice included in the present study. However, causal relationships between network and behavioral changes cannot be determined without more direct manipulations. Instead, we were limited to investigation of correlational relationships (e.g., between the Frontal network and non-sensorimotor behaviors in the RT-treated mice) but found no significant correlations between brain system alterations and cognitive changes.

It is unknown why higher-order brain networks are more vulnerable to whole-brain RT than more primitive sensorimotor networks. Reasons are likely multifaceted, but one possibility is reduced myelination in association cortex relative to primary sensory systems. Cranial irradiation is associated with acute and persistent tissue damage, reduced neurogenesis, oxidative stress, and neuroinflammation, all of which have the potential to contribute to functional network disruptions/reorganization and cognitive dysfunction. Cranial irradiation also robustly affects anatomical substrates required for supporting new and existing functional connections. At the cellular level, several groups have demonstrated changes in synaptic density and dendritic spine complexity following radiation exposure [100]. In the hippocampus, for example, cranial radiation results in decreased dendritic branching and density [101]. The number of spines, spine density, and axonal filopodia (which assist in finding new synaptic targets) are also significantly reduced by whole-brain RT [101, 102]. Dynamic regulation of active synapses is critical for efficient function of existing neuronal circuits while allowing for the formation of new connections following experience and both external and internal demands. Dendritic spines are responsible for neuronal connectivity and are the primary recipients of excitatory input. Changes in spine morphology and/or density following whole-brain RT could affect experience dependent plasticity (e.g., associating auditory tones to painful stimuli) and possibly account for the functional differences we observed related to learning and memory [102]. This explanation is in line with other work showing that radiation compromises neuronal connectivity and memory function through the reduction of presynaptic markers (synaptophysin), spine loss, and diminished long-term potentiation [100, 103].

Chronic changes in brain function following radiation could also reflect dynamic interactions between multiple cell types including neuronal, glial, and endothelial cells. In rats, for example, fractionated whole-brain RT alters neurotransmission and reduces neuronal viability. Initiation of neuronal cell death, inhibition of neurogenesis, and robust astrocytic responses all accompany late radiation-induced changes in brain function [104]. Astrocytes play critical roles in the regulation of neuronal activity, brain metabolism, and cerebral blood flow. While neurons are considered radioresistant, astrocytes are sensitive to gamma irradiation, and RT-induced cognitive impairment is associated with long-term impairment in astrocytic calcium signaling [104, 105]. Additionally, while activated microglia are clearly involved in neuroinflammation, (nonactivated) microglia help to regulate synaptic and structural plasticity during learning and memory. Modern imaging methods and genetic targeting strategies allow future work to examine longitudinal interactions of multiple cell populations over the cortex following whole-brain RT.

The observed divergence between the effects of whole-brain RT on hemodynamic and calcium signaling warrants further investigation. Changes in neurovascular coupling may be a key component of radiation injury, as radiation is known to impact the vasculature and blood brain barrier [36, 37, 106]. Single dose and fractionated whole-brain RT have both been shown to damage brain vasculature via endothelial damage [107]. Radiation dose-dependent effects include suppression of endothelial cell proliferation, endothelial apoptosis, blood-brain barrier disruption, microvascular rarefaction, and breakdown of the extracellular matrix [108]. This vascular compromise significantly affects neuronal function, disturbs the neurovascular unit, and is predictive of cognitive impairment [108–110]. Further work is therefore needed to disambiguate the contribution of whole-brain RT to changes in vascular functioning, purely neuronal signaling, and neurovascular coupling.

## Conclusions

Clinically mimetic whole-brain RT in mice is feasible and effective in eliciting cognitive changes using fraction sizes that are more appropriate for studying the effects of radiation on normal brain tissue. While the cerebral cortex is profoundly expanded in humans compared to mice, our results converge with much of the observed systems-level changes in humans. While broad changes were observed in functional brain network architecture post RT, sensorimotor areas were least impacted, and brain regions overlapping with the mouse default mode network and other association areas were most impacted. Whole-brain RT caused significant performance deficits across a variety of cognitive domains. Our results fill a much-needed gap in understanding the effects of whole-brain RT on functional brain organization, and how network changes may relate to post-treatment cognitive dysfunction. Having established a clinically relevant injury model, future studies can examine therapeutic interventions designed to reduce neuroinflammation-based injury following RT. Given the wide utilization of whole-brain RT in pediatric brain tumor patients, this approach can be used to evaluate functional alterations following whole-brain RT in juvenile mice. Lastly, our future goals are to utilize this platform to study preventative and restorative pharmacologic strategies in order to ameliorate the effects of whole-brain RT on brain organization and function.

### Supplementary information

ESM 1 Figure S1- Functional connectivity (FC) tends to move towards zero three months after whole-brain radiotherapy (RT). Absolute value FC for the mice who received whole-brain RT (RT+3mo) was subtracted from the absolute value FC for the unirradiated mice (RT-). The result is displayed via the grayscale matrix on the left, where colors on the y-axis indicate the functional network to which each row and column corresponds (functional networks are displayed on the brain to the right for reference). Brighter colors in the matrix correspond to higher absolute value FC in the RT- mice, meaning that FC was reduced (moved toward 0) after whole-brain RT. Darker colors correspond to no changes in absolute value FC. Absolute value FC was reduced (moved towards 0) within the Parietal, Somatomotor, Retrosplenial, Posterior Retrosplenial, Primary Visual, Frontal, and Auditory networks and most prominently between the Parietal-Somatomotor, Parietal-Retrosplenial, Parietal-Posterior Retrosplenial, Somatomotor-Retrosplenial, Somatomotor-Posterior Retrosplenial, and Retrosplenial-Posterior Retrosplenial network pairs. (EPS 639 kb)

ESM 2 Figure S2- Sensorimotor behavioral measures after whole-brain RT. **(A)** Overall locomotor activity, **(B)** exploratory rearing behavior, and **(C)** exploration of the center of the test cage were significantly reduced after whole-brain RT. **(D)** Time to climb a vertical screen, **(E)** change in pre-pulse inhibition of the startle response, and **(F)** locomotor activity during the novel object recognition test were not significantly different between groups. (EPS 1187 kb)

## Data Availability

All data will be made available upon reasonable request. Please contact the corresponding author at aqbauer@wustl.edu.

## Appendix A

Absolute FC was computed for each block-average FC matrix (group-level). See the main text Methods Section 2.6 for descriptions of each behavioral test. Effect sizes and statistical significance are reported in the main text Results.

## Data and code availability

All neuroimaging and behavioral testing data will be made freely available.
